# Advances in exosome biomarkers for cervical cancer

**DOI:** 10.1002/cam4.4828

**Published:** 2022-05-16

**Authors:** Zihan Ran, Shaobo Wu, Zijng Ma, Xiuwen Chen, Jing Liu, Jingcheng Yang

**Affiliations:** ^1^ Department of Research Shanghai University of Medicine & Health Sciences Affiliated Zhoupu Hospital Shanghai China; ^2^ Inspection and Quarantine Department, The College of Medical Technology Shanghai University of Medicine & Health Sciences Shanghai China; ^3^ The Genius Medicine Consortium (TGMC) Shanghai China; ^4^ State Key Laboratory of Genetic Engineering, Human Phenome Institute, School of Life Sciences and Shanghai Cancer Center Fudan University Shanghai China; ^5^ Greater Bay Area Institute of Precision Medicine Guangzhou China

**Keywords:** biomarker, cervical cancer, exosomes, microRNA

## Abstract

Cervical cancer (CC) ranks as the fourth most frequently diagnosed malignancy in females worldwide. Exosomes are a subclass of extracellular vesicles released by nearly all types of cells that act as cargo transport vehicles, carrying proteins, and genetic material (such as miRNAs, long noncoding RNAs, and mRNAs) derived from their parent cells may affect receiving cells and thus have emerged as key players in several biological processes, including inflammatory pathways. In this review, we concentrated on the findings of exosome investigations in CC, particularly their components. They direct the actions of CC cells by inducing surface molecules associated with various biological pathways. We summarized the current knowledge of exosomal RNAs and proteins from CC cells and discussed the feasibility of exosomes as potential biomarkers for CC. We suggest that cancer‐derived exosomes promote metastasis in CC by supporting EMT, controlling the proliferation, invasion, or migration of cancer cells, as well as influencing immune escape and aiding angiogenesis. Overall, cancer‐derived exosomes are critical in the progression of CC, and further studies are necessary to advance our understanding of the clinical value of exosomes in CC.

## Graphical abstract

Cancer‐derived exosomes may promote CC metastasis by boosting cellular epithelial‐mesenchymal transformation (EMT), controlling the proliferation, invasion, or migration of cancer cells, as well as influencing immune escape and aiding angiogenesis.

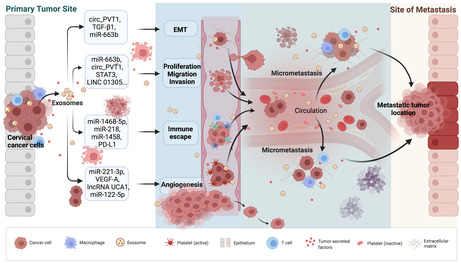

## 1 INTRODUCTION

Cervical cancer (CC) is a type of cancer that originates from cells produced at the squamocolumnar junction of the uterine cervix.^1^ Cervical cancer ranks as the fourth most common cancer among women globally and is one of the leading causes of cancer deaths in women, especially when discovered at an advanced stage.^2^ The development of female CC requires infection with “highly carcinogenic” strains of human papillomavirus (HPV).^3^ Human papillomavirus type 16 is the most common form of HPV associated with CC, followed by HPV18, HPV45, and HPV 31.^4^, ^5^ In the past few years, exosomes have been recognized as a critical factor in several kinds of cancer and other pathologies. They may be used in the clinic as potential biomarkers for detecting and differentiating malignant from noncancerous tissue.^6^, ^7^ To date, thousands of articles have demonstrated the involvement of exosomes in cancer progression.^8^, ^9^ This review focuses on CC, emphasizing the progression of exosomal molecules (RNAs and proteins) in CC and the impact on the immune response. In addition, the feasibility of exosomes as potential biomarkers for CC is discussed. Cancer‐derived exosomes may promote CC metastasis through multiple pathways and deserve in‐depth study.

## 2 STRUCTURE AND FUNCTION OF EXOSOMES

*Bonucci* and *Anderson* first reported exosomes in the late 1960s.^10^, ^11^ These particles are extracellular vesicles with a structure similar to that of cells, ranging from 30 to 150 nm in diameter, and they are present in the same tissue as their associated cells.^12^, ^13^, ^14^ Fluorescence microscopy has been used to detect the presence of exosomes.^15^ Nucleic acids, proteins, lipids, and metabolites have been proposed as biomarkers due to their unique molecular properties (8). Exosomes have potential as biomarkers because of their high contents of cytoskeletal proteins, MHC class I and II proteins, adhesion proteins (tetramers, integrins), and encapsulated nucleic acids.^16^

Delorme‐Axford et al. demonstrated that exosomal miRNA (chromosome 19 miRNA cluster, C19MC) could significantly prevent infection by inducing autophagy and resistance against viral infections, such as poliovirus, human cytomegalovirus, and herpes simplex virus 1.^17^ It has been extensively documented that exosomes play a role in the immunological response. Takahashi et al. demonstrated that human fibroblast exosomes eliminate damaged cytosolic DNA, allowing the cells to maintain their normal state.^18^ Exosomes have the potential to influence the immune response by mesenchymal stem cells and signaling pathways in recipient cells, mainly through the transfer of miRNAs.^19^ Exosomal miRNAs can also circulate between dendritic cells and suppress gene expression, allowing them to function as antigen receptors.^20^, ^21^ In general, exosomes are involved in immune responses not only to cancer cells, but also to infectious agents (bacteria, viruses, fungi, and parasites).

## 3 VARIOUS CARGOES IN EXOSOMES

Exosomes transport various cargoes, including RNAs, proteins, and DNA, which can be picked up directly by other target cells or via biofluids, provoking a variety of phenotypic responses.

### 3.1 Exosomes as RNA carriers

Exosomes contain a diverse variety of RNA sequences, implying the presence of several RNA biotypes.^22^, ^23^ Exosomal influence is governed not only by their origin, but also by their cargo composition. Numerous studies have identified the bulk of known ncRNA biotypes, including small nuclear RNAs (snoRNAs), rRNAs, long noncoding RNAs (lncRNAs), PIWI‐interacting RNAs (piRNAs), and transfer RNAs (tRNAs).^24^ Exosomes are found in numbers of approximately 2000 trillion in normal human blood and up to 4000 trillion in tumors.^25^ In recent years, noncoding RNAs have been extensively studied and have been revealed to function in distant tissues, implicating them in various biological processes via various mechanisms, including epigenetic modifier protein recruitment, mRNA decay regulation, and translation.^26^, ^27^

### 3.2 Exosomes as protein carriers

Exosomes are essential protein carriers. They include a varied array of transmembrane proteins, lipid‐anchored membrane proteins, peripherally associated membrane proteins, and exosome lumen‐soluble proteins.^28^ Additionally, exosomes carry surface proteins involved in peripheral nervous system communication, the majority of which are engaged in cell‐to‐cell communication.^29^, ^30^ These surface proteins include tumor necrosis factor (TNF) and wingless (Wnt) proteins.^31^, ^32^ Thus, exosomes can impact the activity of their target cells directly or indirectly via membrane and surface proteins.

### 3.3 Exosomes as DNA carriers

Exosomes can transfer large amounts of DNA from cells.^18^ Single‐stranded, double‐stranded, and genomic DNA are all types of exosomal DNA.^33^, ^34^ Due to the encapsulation of exosome serum DNA, it is more stable than unencapsulated DNA in a range of storage conditions.^35^ According to Liang Wang et al., exosomal double‐stranded DNA may be used as a biomarker for pheochromocytoma and paraganglioma diagnosis.^34^ Thakur et al. found that the capacity of exosomal DNA to identify mutations in parental cancer cells demonstrates its enormous therapeutic potential as a circulating cancer biomarker.^36^ However, the amount of exosomal DNA present within the organelle is unclear. Further research will be needed in the future to determine how DNA enters exosomes and how exosomal DNA may be utilized to aid in the identification and treatment of cancer.

## 4 EXOSOMES IN OTHER CANCERS

### 4.1 Exosomes in lung cancer

Lung cancer is a diverse illness with various subgroups with pathological and clinical significance. It is one of the China's most common malignant tumors, ranking first in cancer‐related mortality.^37^, ^38^ Because of inadequate diagnosis and prognosis at an early stage, the mortality rate for lung cancer patients remains high.^39^ Therefore, identifying novel targets and biomarkers as practical tools for the management of lung cancer represents a significant challenge.

Numerous studies have focused on the critical role of lung cancer cell exosomes in the occurrence, early detection/diagnosis, and drug resistance of lung cancer. JAE YOUNG KIM et al. discovered that a large amount of COX‐2 loaded on the exosomes of lung cancer cells, which could then be transferred to neighboring or distant cells.^40^ Nan Zhang et al. found that the exosome‐derived circSATB2 protein plays a vital role in advancing non‐small cell lung cancer (NSCLC). Through direct binding to miR‐326, CircSATB2 may modulate FSCN1 expression in NSCLC cells, hence boosting cell proliferation, migration, and invasion in the tumor environment.^41^ Following the findings of a study conducted by Lingyu Li and colleagues, serum exosomal FECR1 may be a valuable biomarker for diagnosing the progression of small cell lung cancer (SCLC) in patients.^42^

### 4.2 Exosomes in liver cancer

Liver cancer is the second leading cause of cancer‐related death worldwide.^43^ Among types of liver cancer, hepatocellular carcinoma (HCC) is the most common. Chronic infection with hepatitis B virus (HBV) is a crucial risk factor for the development and progression of hepatocellular carcinoma (HCC), accounting for more than half of all HCC cases worldwide.^44^

Identifying the involvement of exosomal miRNAs and proteins throughout the development of HCC is essential. Many investigations on human samples and several experimental models have been carried out. Research studies have discovered that increased levels of miR‐122 and miR‐99 family expression in exosomes may promote HBV replication.^45^, ^46^ According to one study, higher miR‐199‐3p and miR‐201 expression levels in exosomes may be associated with decreased HBV replication.^47^

Recent research has demonstrated that exosomal miRNAs may be beneficial in the diagnosis and prognosis of HCC. According to the findings of these studies, high levels of let‐7, miR‐122, and miR‐125a‐5p expression were associated with the development of HCC.^48^, ^49^, ^50^ Exosomal miR‐638, miR‐296, miR137, and miR‐940 expression levels were found to be associated with the poor prognosis of HCC patients, and these findings have been confirmed in other cancers.^51^, ^52^, ^53^, ^54^ These findings indicate that exosomal miRNAs may contribute to the diagnosis and prognosis of HCC patients.

### 4.3 Exosomes in gastric cancer

Gastric cancer (GC) is one of the most common malignant tumors globally and has high morbidity and mortality. GC is much more prevalent in Asian countries than in non‐Asian countries.^2^ Infection with *Helicobacter pylori*, high salt intake, and eating fewer fruits and vegetables are risk factors for gastric cancer.^55^ Therapy for GC has made significant progress in recent decades, and numerous attempts have been undertaken to develop successful treatment solutions for the condition. However, the morbidity and mortality associated with GC are still high. Therefore, identifying new targets and biomarkers as practical tools for the management of gastric cancer is a significant problem in today's world.

When comparing patients with T4 stage cancer to those with T1 to T3 stage cancer, Tokuhisa et al. discovered that patients with T4 stage cancer had greater exosomal miR‐21 and miR‐1225‐5p expression levels.^56^ CD97 proteins are tetraspanins found on the surface of exosomes and in the cell membrane. Exosomes derived from cells with high or low CD97 expression can stimulate the proliferation and migration of carcinoma cells by activating the exosome‐mediated MAPK signaling pathway.^56^ According to Chao Li et al., exosomal miRNAs may also play a role in activating this pathway.^57^ Furthermore, they discovered that CD97 promotes the proliferation and migration of gastric cancer cell lines in vitro.^57^

## 5 ROLES OF EXOSOMES IN CC

### 5.1 Exosomes in CC immunity

Exosomes released by both nonimmune and immune cells play a critical role in the immune regulation of CC cells.^58^ Buschow SI et al. discovered visible peptide–MHC class II complexes on the surfaces of exosomes, which demonstrated the involvement of exosomes in intercellular antigen transfer.^59^ Furthermore, cancer‐derived exosomes have been shown to stimulate immune responses in laboratory animals.^60^

Natural killer (NK) cells play critical roles in antiviral and anticarcinogenic immune responses in HPV‐related malignancies.^61^ Apart from cytotoxic effects, NK cells are capable of secreting a large and diverse array of signaling molecules in response to the cytokine pattern existing in the tumor environment.^62^ Due to the critical function of NK cells in the immune response to HPV, this cell type has been extensively studied in HPV‐related carcinogenesis, particularly in CC. Since the actions of NK cells are dependent on the receptors and ligands linked with them, numerous studies have examined the impact of these molecules on cervical carcinogenesis. Jimenez‐Perez et al. postulated that CC cells might generate decreases in the amounts of NKG2D and NKp46 on the surfaces of NK cells, which were connected with a decrease in cytotoxic effects.^63^ According to Wen‐Chun Chang et al., Treg cell function significantly decreased NKG2D expression in CC patients.^64^

In both ex vivo and in vivo studies, it has been demonstrated that dendritic cell (DC)‐derived exosomes (Dexo) can induce a specific antitumor immune response.^60^ Dexo carries immunologic molecules capable of inducing an intense tumor cytotoxic response.^65^ Poly(I: C) was found to have a substantial auxiliary effect on immune responses in some research studies.^66^ Shisheng Chen et al. established that incubation with poly(I: C) during exosome synthesis dramatically increased the anti‐CC properties of Dexo(E7 + pIC).^60^ They also discovered that Dexo significantly slowed the progression of tumors in mice.^60^

In general, exosomes play a key role in the immune response to CC. Exosomes derived from CC may promote cancer progression by impairing NK activities. Exosomes can also produce a targeted antitumor immune response by interacting with dendritic cells.

### 5.2 Exosomal RNAs in CC

The first report about exosomal RNAs was made by Ratajczak J et al.,^67^ who claimed that exosomes contain RNAs, which can transfer extracellular RNAs to other cells or organs in a functional form.^67^ Small noncoding RNAs (ncRNAs) are particularly abundant in exosomes that contain disproportionate amounts of small nuclear RNAs (snRNAs), microRNAs (miRNAs), Y RNAs, or fragmented ncRNAs.^68^, ^69^ However, most CC studies have focused on exosomal miRNAs.^29^, ^70^, ^71^, ^72^

MiRNAs are single‐stranded RNAs with a length of 22 nucleotides and are found in various organisms.^73^ MiRNAs are vital in the regulation of gene transcription.^74^, ^75^ As guides in posttranscriptional gene silencing, miRNAs can cause snippet mRNA breakage or translational repression, which affects cellular appearance and function.^76^, ^77^ Several miRNAs have been discovered to regulate B‐cell differentiation,^78^ antiviral defense,^79^ and carcinogenesis in animals.^80^ Therefore, miRNAs are important regulators of cell signaling and homeostasis in the tumor microenvironment.^81^ It is possible to assess the expression of miRNAs, which can be utilized as diagnostic or prognostic biomarkers in CC. Lui et al. published the first research to compare the variation of two miRNAs (miR21 and miR‐143) in female CC cell lines and normal cervical tissues.^82^ They found that the expression levels of miR‐21 and miR‐143 in CC cells were different from those in normal cervical tissue.^82^ Genetic alterations of miRNA loci, such as point mutations and even epigenetic silencing, including DNA methylation or dysregulation of miRNA integrators and transposable elements, have all been linked to anomaly‐induced miRNA expression.^83^ The levels of RNA expression in cervical neoplasia biopsy samples and sera from females with CC have been the subject of numerous studies (Table 1).

**TABLE 1:** RNAs derived from CC cell exosomes

Type	Expression level	Clinical value	Cargo	Reference
miRNA	Up	Diagnosis/therapy	miR‐221‐3p	132
miRNA	Down	Diagnosis	miR‐30d‐5p, let‐7d‐3p	72
lncRNA	Up	Diagnosis	HOTAIR	135
lncRNA	Up	Predictive	lncRNA DLX6‐AS1	
miRNA	Up	Diagnosis/prognostic	miR‐196a	
miRNA	Up	Diagnosis/therapy	miR‐486‐5p	
miRNA	Down	Diagnosis	miR‐125a‐5p	
lncRNA	Up	Therapy	LINC01305	125
miRNA	Up	Diagnosis	miR‐221/222	136
miRNA	Up	Diagnosis	miR‐21, miR‐146a	137
miRNA	Up	Therapy	miR‐22	138
lncRNA	Down	Diagnosis/predictive	MEG3, PVT1	139
lncRNA	Down	Therapy	GAS5	
lncRNA	Down	Therapy	lncRNA STXBP5‐AS1, TUSC8	
lncRNA	Down	Predictive/prognostic	XLOC_010588	
lncRNA	Up	Predictive	LncRNA SPRY4, ZEB1‐AS1, LINC01305, LOC105374902	
miRNA	Down	Diagnosis/therapy	miR‐34b	140
miRNA	Up	Therapy	miR‐106a/b	141
miRNA	Up	Diagnosis	miR‐146a‐5p, miR‐151a‐3p, miR‐2110, miR‐21‐5p	142
circRNA	Up	Therapy	circRNA‐PVT1	129
lncRNA	Up	Therapy	lncRNA HNF1A‐AS1	143
miRNA	Up	Therapy	miR‐221‐3p	130
miRNA	Up	Therapy	miR‐9	144
miRNA	Up	Diagnosis	miRNA‐20a, miRNA‐203, miRNA‐21, miRNA‐205, miRNA‐218, miR‐485‐5	145
miRNA	Up	Diagnosis / Therapy	miR‐7, miR‐10a, miR‐17‐5p, miR‐135b, miR‐149, miR‐203	
miRNA	Up	Diagnosis/therapy	miR‐877‐3p	146
miRNA	Up	Diagnosis/therapy	miR‐155‐5p	147
miRNA	Up	Therapy	miR‐663b	127
miRNA	Down	Therapy	miR‐5590‐3p, miR‐3156‐3p, miR‐10b	148
miRNA	Down	Therapy	miR‐34a, miR‐1284, miR‐142	58
miRNA	Down	Therapy	miR‐429	
miRNA	Down	Therapy	miR‐101	
miRNA	Down	Therapy	miR‐34a, miR‐1284, miR‐142	
miRNA	Down	Diagnosis/therapy	miR‐24, miR‐451, miR‐125a	
miRNA	Up	Diagnosis/therapy	miR‐130a	
miRNA	Up	Diagnosis/therapy	miR‐155	

### 5.3 Exosomal proteins in CC

**FIGURE 1:**
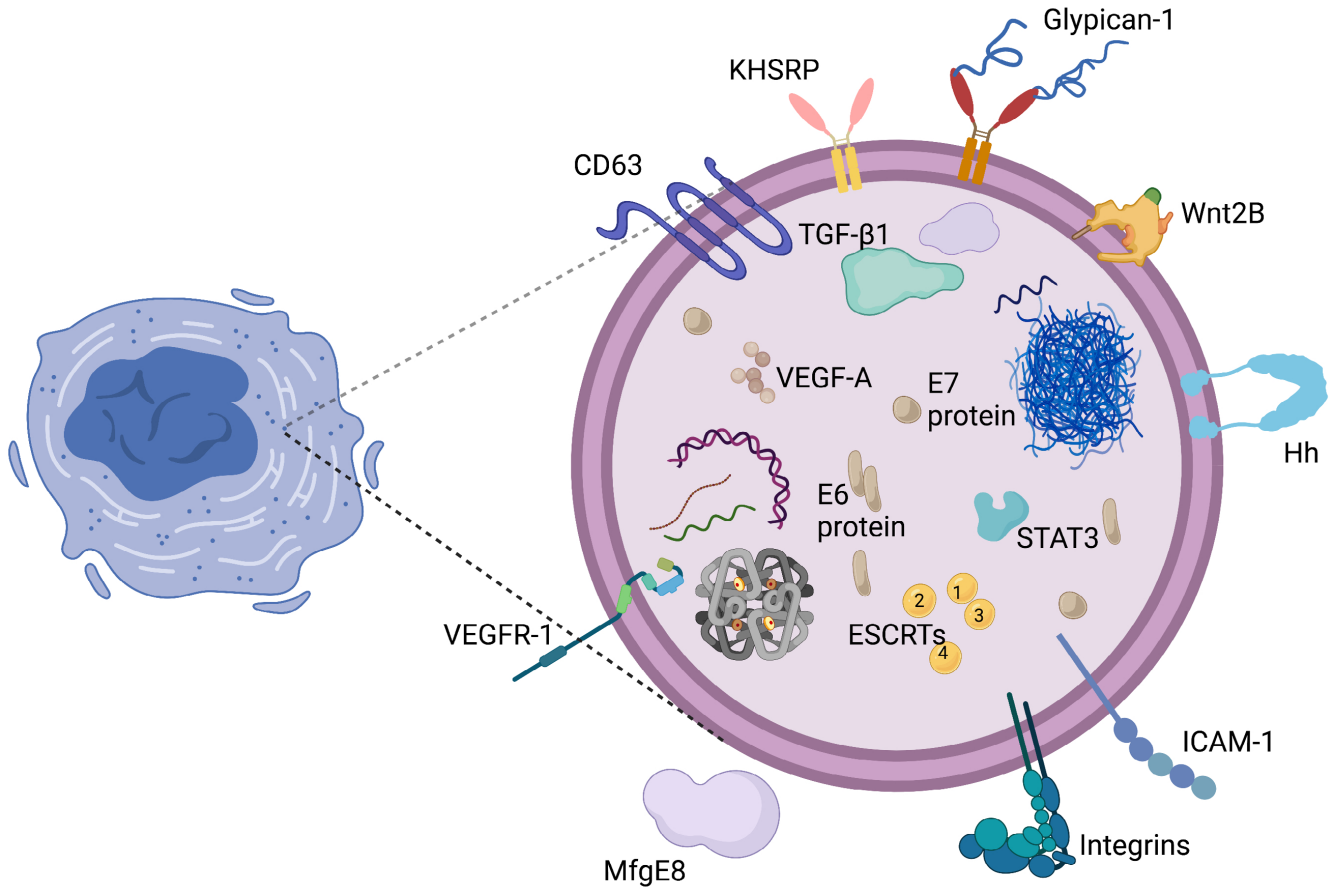
Structural characteristics of CC‐derived exosomes. CC‐derived exosomes are a subtype of extracellular vesicles that range in diameter from 30 to 200 nm and are abundant in certain proteins, lipids, nucleic acids, and glycoconjugates. Objects depicted in the figure are illustrative rather than exhaustive. Abbreviations: KHSRP, KH‐type splicing regulatory protein; Wnt2B, wingless protein 2B; Hh, Hedgehog; TGF‐β1, transforming growth factor‐beta 1; VEGF‐A, vascular endothelial growth factor A; STAT3, signal transducer and activator of transcription 3; E6 protein, a protein encoded by human papillomavirus; E7 protein, a protein encoded by human papillomavirus; VEGFR1, vascular endothelial growth factor receptor 1; MfgE8, milk fat globule protein E8; ESCRTs, endosomal sorting complexes required for transport; ICAM‐1, intracellular adhesion molecule‐1.

There are several types of transmembrane and lipid‐anchored membrane proteins in exosomes.^28^, ^84^ Exosome proteins are peripherally linked with membrane proteins and soluble proteins in the exosome lumen (Figure 1). This section will provide an overview of many of the more relevant and intriguing proteins consistently identified in CC exosomes.

HPV pathogenesis was initially reported in 2009, and extracellular vesicles were found to function in this pathogenesis.^85^ Following this finding, the same author verified the presence of extracellular survivin within exosomes.^85^ Several studies suggest that specific exosomal proteins are involved in the progression of CC,^86^, ^87^, ^88^ including metastasis, invasion, and resistance to chemotherapy.^89^

ATF1 is a transcription factor protein that is involved in cell development, survival, and other biochemical processes.^90^ Overexpression of ATF1 has been observed in nasopharyngeal carcinoma (NPC), gastrointestinal clear cell sarcoma, and other cancers in various investigations.^91^, ^92^, ^93^ Interestingly, Yanhua Shi et al. discovered that ATF1 levels in the blood exosomes of a CC mouse model were dramatically elevated.^94^ Therefore, it should be no surprise that high ATF1 expression in exosomes might be used as a potential diagnostic biomarker for CC.

Small GTPases known as RAS proteins control cell proliferation, survival, and differentiation by acting as downstream effectors of growth factor receptor signaling.^95^ When CC mouse blood exosomes were compared to normal mouse tissues in Yanhua Shi's study, the level of RAS protein expression was more than five times higher.^94^ The RAS gene confers a substantial positive predictive value for CC. In addition, the RAS oncogene is mutated in approximately 35% to 45% of colorectal cancers, depending on the type.^96^ Consequently, it is necessary to determine whether detection of a mutated RAS gene should be part of the diagnostic process.

Numerous studies have examined the protein expression levels in cervical neoplasia tissues and serum from CC patients, and the results are promising (Table 2). Early detection and prevention of CC are essential, and the exosomal detection approach may prove to be an effective tool for the early diagnosis of CC in the future.

**TABLE 2:** Proteins derived from CC cell exosomes

Type	Clinical level	Clinical value	Cargo	Reference
Protein	Down	Diagnosis/therapy	THBS2	132
Protein	Up	Diagnosis/therapy	P13k, Akt, mTOR	117, 149
Protein	Up	Diagnosis/therapy/prognosis	Wnt‐2b	116
Protein	Up	Therapy	MUC16, SIRPA, E7	150
Protein	Up	Therapy	CHMP4B, STX‐7, RPL28	151
Protein	Up	Therapy	PTCH1, GLI1	118
Protein	Down	Therapy	MAPK10	136
Protein	Up	Diagnosis	ATF‐1, RAS	100
Protein	Down	Therapy	HPGD	152
Protein	Up	Therapy	STAT3	153
Protein	Down	Therapy/prognosis	IFNAR1	154
Protein	Up	Diagnosis	E6, E7	155
Protein	Up	Therapy	KHSRP	131
Protein	Down	Therapy	ISG15	156
Protein	Up	Diagnosis	E2(NF‐κB)	157
Protein	Up	Therapy	E5(COX‐2)	158

## 6 EXOSOMES AS A BIOMARKER IN CC

Biological markers (also known as biomarkers) are molecules that are used to diagnose and/or predict the outcome of certain diseases.^97^ Since exosomes are relatively stable, they can be found in blood, urine, and other body fluids using inexpensive, straightforward, sensitive, and reliable procedures even after years of sample preservation.^98^ In the era of precision medicine, exosomes could be a new class of biomarkers for cancer diagnosis, treatment, and prognosis.

### 6.1 Exosomes as a diagnostic or therapeutic biomarker

Exosomal miRNA‐containing complexes have the potential to exert various therapeutic effects. In particular, it is possible to envision collecting exosomes from healthy donors and injecting them into patients to treat illness.^99^, ^100^

Some studies have demonstrated that miR21 enhances cancer propagation, inflammation, and translation.^101^, ^102^, ^103^, ^104^ In CC, upregulated miR21 targeted PDCD4, PTEN, RASA1, and TIMP3.^105^ Yang et al. discovered that miR181b reduced the generation of cyclic adenosine monophosphate (cAMP) in CC cell lines by downregulating adenylyl cyclase 9 expression to prevent apoptosis and promote cell proliferation.^106^ According to the findings of another study, miR143 may affect the death and proliferation of HeLa cells in CC by targeting the transcription factor HIF1.^107^ In addition, the overexpression of miR21 has been shown to influence the survival, proliferation, and invasiveness of CC cells, which may be accomplished by targeting tissue inhibitor of metalloproteinase 3 (TIMP3).^108^, ^109^

As with exosomal ncRNAs, exosomal proteins or transmembrane proteins also play important roles in the diagnosis or therapy of CC.^5^, ^110^ Liang lj et al. demonstrated that CC‐derived exosomal Wnt2B could drive fibroblast activation into cancer‐associated fibroblasts, and this discovery points the way forward in developing diagnostic and therapeutic targets for CC progression.^111^ Zhang W et al. reported that the PI3k/Akt/mTOR signaling pathway might provide possible diagnostic biomarkers or therapeutic targets via exosomes isolated from vaginal secretions.^112^ In addition, exosomes can promote cervical angiogenesis via the hedgehog–GLI signaling pathway, identifying exosomes as prospective therapeutic targets for locally progressed metastatic cervical lesions.^113^

These studies indicate that exosomes might promote the progression of CC through proteins or RNAs in exosomes, which may play a critical role in the diagnosis or therapy of CC.

### 6.2 Exosomes as a prognostic biomarker

CC is known to be associated with HPV infection, which is the major risk factor for the disease. The continuous expression of the HPV oncogenic proteins E6 and E7 has been related to the progression of CC through the degradation of the tumor suppressor protein p53 and the deactivation of the retinoblastoma protein (pRB).^114^, ^115^, ^116^, ^117^, ^118^ Exosomes also play important roles in the development and progression of CC. For example, the overexpression of miRNA‐944 seems to be a biomarker of an adverse prognosis in advanced CC cases.^119^ A study conducted in the advanced FIGO stage found that the group with high exosomal miR‐664 expression survived for a shorter period than the group with low exosomal miR‐664 expression.^120^ Consequently, exosomes may potentially be used as prognostic biomarkers for CC patients.

Exosomes, which transport biomolecules from one cell to another, are important mediators of cellular communication. Exosomal ncRNAs are engaged in various cellular and biological processes, such as cellular development, cellular differentiation, and migration.^121^ Due to their outstanding level of stability, exosomes may be detected in the bloodstream, making serum exosomal ncRNAs an intriguing prospective biomarker in many malignancies, including CC.^122^, ^123^ According to the findings of one study, the serum exosomal lncRNA DLX6‐AS1 level in CC patients was considerably higher than the levels in CIN abnormal and normal cases.^122^ Moreover, DLX6‐AS1 may accelerate the progression of CC by sponging miR‐16‐5p and upregulating ARPP19, which offers novel insight into the prognosis and remedy of CC.^124^ In addition, the overexpression of LINC01305 or knockdown significantly increased or decreased the development of CC,^125^ which indicated that a higher LINC01305 level was associated with a worse prognosis. Furthermore, BBOX1‐AS1 mediates HOXC6 expression via miR‐361‐3p and HuR, promoting CC development,^126^ and BBOX1‐AS1 overexpression predicts a worse prognosis for CC cases.

Although exosomes in the blood of CC patients have not been adequately studied, current experimental evidence supports the notion that these ncRNAs can be used to develop, improve, or strengthen CC prognosis and management strategies.

### 6.3 Cancer‐derived exosomes promote CC metastasis

Since exosomes can carry biomolecules for intercellular transmission, the content of exosomes (such as ncRNAs or proteins) contributes to the metastasis of CC to a certain extent. We can find some evidence of this influence in several recent studies. CC exosomal miRNA‐663b, cicr_PVT1 and TGF‐β1 are related to CC epithelial‐mesenchymal transition (EMT).^89^, ^127^ In addition, CC‐derived exosomal miRNA‐663b, miRNA‐221‐3p, miRNA‐146b‐3p, miRNA‐125a, cicr_PVT1, wnt‐2b, and miRNA‐744 are critical in the proliferation, invasion, and migration of CC cells.^89^, ^111^, ^128^, ^129^, ^130^ Additionally, exosomal miRNA‐221, miRNA‐1468‐5p, lincRNA UCA1, and VEGF‐A play crucial roles in CC immune escape and promote angiogenesis.^113^, ^130^, ^131^, ^132^, ^133^

**FIGURE 2:**
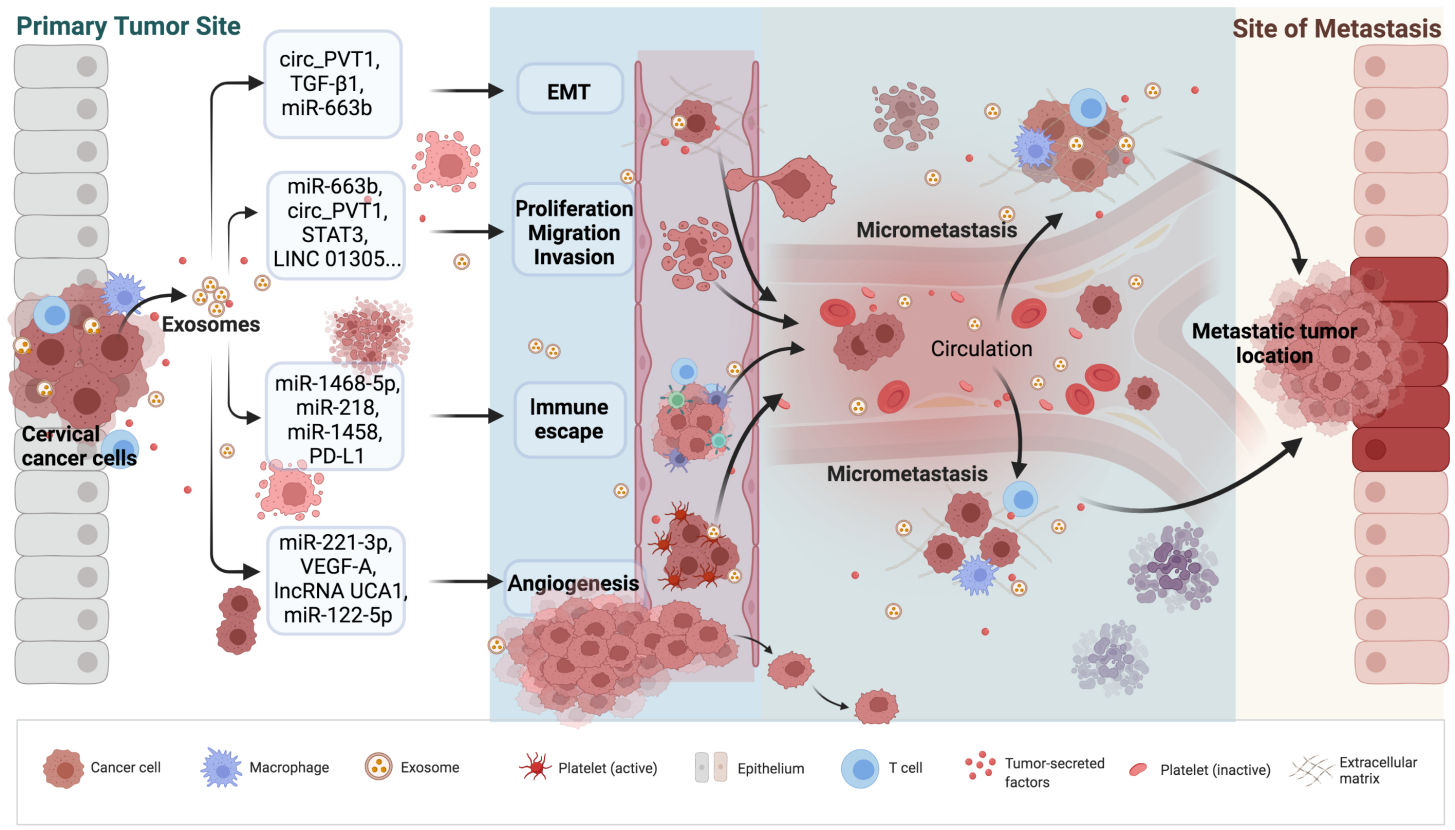
Cancer‐derived exosomes promote CC metastasis. Activated CC‐derived exosome signaling pathways interact with adjacent cells to enhance CC development by boosting cellular epithelial‐mesenchymal transformation (EMT), controlling cell proliferation and migration, and modulating immune escape and angiogenesis.

Based on these studies, we suggest that cancer‐derived exosomes promote metastasis in CC by supporting EMT, controlling the proliferation, invasion, or migration of cancer cells, influencing immune escape, and aiding angiogenesis (Figure 2). Although the roles of exosomes in the most critical and fundamental connections involved in CC metastasis remain unknown, this is a very worthwhile direction for in‐depth research that requires additional investigation.

## 7 CONCLUSIONS AND PROSPECTS

In recent years, exosomes and their function in cancer have become the subject of increasing research studies. Exosomes derived from cancer can have antagonistic effects on the immune response to malignancies.^134^ They can aid tumor cells in evading immune surveillance and developing immunological tolerance, whereas exosomes generated from immune cells have been shown to impede tumor cell growth, proliferation, and metastasis.^58^

Exosomes carry many functional components and play roles in a wide range of physiological and pathological processes in the body. In this review, we concentrated on the findings of exosome investigations, specifically on the primary components of exosomes, which include many RNAs and proteins that direct the actions of CC cells by inducing surface molecules linked to various biological pathways. The appropriate transport EVs for transcripts, proteins, and ncRNAs, exosomes may be used as diagnostic, therapeutic, or prognostic biomarkers in CC. Furthermore, cancer‐derived exosomes can influence CC signal transduction pathways by interacting with various mechanisms involved in tumor origin, progression, and metastasis. This capacity enables the development of a plethora of novel methods for cancer detection and therapy. Obtaining liquid biopsies from patients is fairly straightforward. Thus, exosomes will increasingly be studied to aid in early cancer detection. In addition, the ability of exosomes to modulate immune responses in the cancer environment can be used to develop safe and reliable tumor vaccines.

## CONFLICT OF INTEREST

The authors declare that they have no conflict of interest.

## AUTHOR CONTRIBUTIONS

ZR and SW, the main author of the study, conceived the study and contributed to writing and editing. JY and ZR took part in designing and conducting the study. ZM and XC curated the exosome‐related data in the literature. JL reviewed and revised the manuscript. All of the authors read and approved the final manuscript.

## Data Availability

Data sharing not applicable to this article as no datasets were generated or analysed during the current study.
